# Pathogen blocking in *Wolbachia*-infected *Aedes aegypti* is not affected by Zika and dengue virus co-infection

**DOI:** 10.1371/journal.pntd.0007443

**Published:** 2019-05-20

**Authors:** Eric P. Caragata, Marcele N. Rocha, Thiago N. Pereira, Simone B. Mansur, Heverton L. C. Dutra, Luciano A. Moreira

**Affiliations:** Grupo Mosquitos Vetores: Endossimbiontes e Interação Patógeno-Vetor, Instituto René Rachou—Fiocruz, Belo Horizonte, MG, Brazil; International Centre for Genetic Engineering and Biotechnology, INDIA

## Abstract

**Background:**

*Wolbachia*’s ability to restrict arbovirus transmission makes it a promising tool to combat mosquito-transmitted diseases. *Wolbachia*-infected *Aedes aegypti* are currently being released in locations such as Brazil, which regularly experience concurrent outbreaks of different arboviruses. *A*. *aegypti* can become co-infected with, and transmit multiple arboviruses with one bite, which can complicate patient diagnosis and treatment.

**Methodology/principle findings:**

Using experimental oral infection of *A*. *aegypti* and then RT-qPCR, we examined ZIKV/DENV-1 and ZIKV/DENV-3 co-infection in *Wolbachia*-infected *A*. *aegypti* and observed that *Wolbachia*-infected mosquitoes experienced lower prevalence of infection and viral load than wildtype mosquitoes, even with an extra infecting virus. Critically, ZIKV/DENV co-infection had no significant impact on *Wolbachia*’s ability to reduce viral transmission. *Wolbachia* infection also strongly altered expression levels of key immune genes *Defensin C* and *Transferrin 1*, in a virus-dependent manner.

**Conclusions/significance:**

Our results suggest that pathogen interference in *Wolbachia*-infected *A*. *aegypti* is not adversely affected by ZIKV/DENV co-infection, which suggests that *Wolbachia*-infected *A*. *aegypti* will likely prove suitable for controlling mosquito-borne diseases in environments with complex patterns of arbovirus transmission.

## Author summary

*Wolbachia* is an endosymbiotic bacterium that infects insects. It has been artificially transferred into *Aedes aegypti*, a mosquito species that can transmit medically important viruses including dengue, chikungunya, and Zika. *Wolbachia* in *A*. *aegypti* limits infection with these viruses, making the mosquitoes much less capable of transmitting them to people. In tropical areas, where these viral pathogens are commonly found, it is not unusual for outbreaks of different viruses to occur at the same time, which can complicate diagnosis and treatment for those afflicted. Mosquitoes with *Wolbachia* are currently being released into these areas to reduce transmission of these diseases. In our study, we assessed whether *Wolbachia* infection in *A*. *aegypti* mosquitoes could still effectively inhibit the dengue and Zika viruses if the mosquitoes were fed both viruses at the same time. We found that *Wolbachia* was still very effective at inhibiting the replication of both viruses in the mosquito, and likewise still greatly reduced the chance of transmission of either virus. Our results suggest that *Wolbachia*-infected mosquitoes should be able to limit infection with more than one virus, should they encounter them in the field.

## Introduction

Arboviruses, viruses transmitted by arthropods including *Aedes* mosquitoes, cause diseases that represent a serious threat to human health, with more than half of the global population at risk [1, 2]. Evidence suggests that the impact of these diseases is growing, as there has been an increase in case numbers of established arboviral infections [1, 3–5], while several other arboviruses have emerged as potential disease agents of the future [6, 7]. Consequently, finding a means to reduce the impact of these diseases is a significant public health issue.

The most significant viral pathogen transmitted by mosquitoes is dengue virus (DENV), which can cause severe fever, and produces an estimated 390 million infections per year [1]. DENV is highly prevalent in Latin America, and in South and Southeast Asia [3]. There are four known DENV serotypes (DENV-1 through DENV-4), and a subsequent infection with a second serotype can produce far more severe symptoms [1, 8]. This is particularly problematic, given that the multiple serotypes often co-circulate [9–11]. Compounding this issue is the lack of adequate treatment for dengue infections. There is no antiviral therapy, and while a vaccine effective against all four serotypes has been involved in late-stage clinical trials, the results have proven controversial [12, 13].

Another notable arbovirus is Zika virus (ZIKV), which was first isolated from febrile sentinel monkeys in 1947, in the Ziika forest of Uganda, and then emerged from relative obscurity to cause a massive outbreak that peaked during 2015–2016 [14]. The majority of cases of ZIKV appear to be asymptomatic, however infection has been associated with significant clinical consequences, including microcephaly and Guillain-Barré syndrome [15–17].

The mosquito *Aedes aegypti* is an important vector of arboviruses including DENV, chikungunya virus (CHIKV), yellow fever virus (YFV), and ZIKV. It has a broad distribution across tropical and sub-tropical regions around the world, where it lives in close association with human dwellings, and typically lays eggs in discarded containers that collect rainwater [18, 19]. Adult female *A*. *aegypti* feed on human blood in order to produce eggs, and they can become infected with an arbovirus after biting someone who is viremic. Within the mosquito, the virus invades the cells of the midgut epithelial layer where it exploits a range of host factors in order to replicate, and then releases mature virions that eventually infect the salivary glands [20–22]. This process takes approximately 7–14 days, depending on intrinsic and extrinsic factors including mosquito genetic background and environmental temperature, after which the mosquito can transmit the virus to new people when it bites them.

With the exception of yellow fever and Japanese Encephalitis virus, there are no effective commercially available vaccines against mosquito-borne arboviral diseases, and there are few viable treatment options available for those infected [1, 2]. For these reasons, disease prevention strategies are usually focused on mosquito control, relying on population elimination by clearing breeding sites, and using chemical insecticides to quickly and effectively kill mosquitoes [23]. However, maintaining persistent control of mosquito populations is a difficult and costly prospect, which has led to the development of many novel mosquito control strategies [24].

One such strategy involves *Wolbachia pipientis*, a maternally inherited bacterial endosymbiont of arthropods and worms that naturally occurs in at least 40% of terrestrial insect species [25]. Stable, heritable *Wolbachia* infections in *A*. *aegypti* have been generated by transinfection, the injection of *Wolbachia* from a donor species into *A*. *aegypti* eggs [26–29]. These *Wolbachia*-infected mosquitoes have been deployed in the field in multiple countries that experience high, endemic DENV transmission, as part of the World Mosquito Program (https://www.worldmosquitoprogram.org) [30–32].

*Wolbachia* is suitable for the biological control of mosquitoes because infection alters mosquito physiology and reproductive biology in ways that make them less effective vectors, facilitating the spread of the bacteria into wild populations [30, 33]. This ability to spread is due to cytoplasmic incompatibility, a reproductive manipulation that acts as a natural form of genetic drive specific to *Wolbachia*-infected insects [34, 35]. Cytoplasmic incompatibility occurs because the mating of *Wolbachia*-infected males to *Wolbachia*–uninfected females does not result in viable progeny. In contrast, *Wolbachia*-infected females produce viable, *Wolbachia*-infected progeny when mating to any male, thereby, proportionally increasing the number of *Wolbachia*-infected individuals.

Infection with some *Wolbachia* strains causes pathogen blocking—the restriction of viral infection and replication in the tissues and reduced likelihood of transmission in *Wolbachia*-infected mosquitoes. *Wolbachia* in *A*. *aegypti* strongly inhibits infection with viruses harmful to humans such as DENV, CHIKV, Mayaro virus, West Nile virus, YFV, and ZIKV [28, 36–42], and has been demonstrated to make them less effective vectors for a range of medically important mosquito-transmitted viruses [43, 44]. Multiple interactions between the mosquito immune system and *Wolbachia* have been characterized, however it is still unclear if any of these are definitively involved in virus blocking [33, 45]. *Wolbachia* may stimulate the host immune system to respond more effectively to viral infection, as the endosymbiont causes broad-spectrum immune induction [46–48]. It notably increases the expression of many immune genes, including antimicrobial peptides [47–50]. It also induces the production of reactive oxygen species, a key factor in antimicrobial immunity [47]. There are also putative links between *Wolbachia* and the mosquito RNAi immune pathway, through the gene Argonaute 2 [51]. *Wolbachia* infection alters miRNA production, and the subsequent changes to gene expression could impact on viral infection [52]. It causes the downregulation of an insulin receptor that is linked to DENV infection [53]. Finally, there is evidence that *Wolbachia* preferentially utilizes host resources, like cholesterol, that are also required for viral infection [54, 55].

Releases of *A*. *aegypti* mosquitoes infected by the *w*Mel *Wolbachia* strain have been ongoing in Rio de Janeiro, Brazil since 2014 (https://tinyurl.com/WMPBrazil, [56]. Mosquito control strategies in disease-endemic regions, including Rio de Janeiro, may be complicated by the co-circulation of different arboviruses [9, 57]. Multiple studies have demonstrated that humans can become infected with more than one mosquito-transmitted virus at a time, complicating diagnosis and recovery [10, 58]. Recent studies have also determined that *A*. *aegypti* can harbour and transmit more than one virus, after experimental co-infection, suggesting that a single mosquito bite could lead to a person contracting two diseases [59, 60]. Interestingly, co-infection may lead to competition between viruses, producing differential infection rates, and even altering transmission rates. For instance, during co-infection with ZIKV/DENV-2, the transmission of ZIKV is favoured [59]. Other studies have shown that co-infection can promote viral infection and transmission. For example, sequential infection with ZIKV and CHIKV enhances ZIKV transmission in *A*. *aegypti* [61], and co-infection of CHIKV/DENV-2 leads to increased replication of DENV, compared to mono-infection [62]. Little is known about the impact of co-infection on the mosquito immune system, however there are differences in the host proteomic profile for CHIKV/DENV co-infected and mono-infected mosquitoes [63], which suggests that there is some impact.

However, to the best of our knowledge, no studies have described the effects of viral co-infection in *Wolbachia*-infected mosquitoes. Given that the mechanism of *Wolbachia*-induced pathogen blocking may be immune based, we were interested to see how the bacterium responded to infection with multiple arboviruses. Any decrease of blocking activity would be particularly important given that *Wolbachia*-infected mosquitoes are currently being released into areas where multiple medically important arboviruses are transmitted. For these reasons, we sought to determine whether co-infection with ZIKV and DENV-1, or with ZIKV and DENV-3, all recently circulating in Brazil, would lead to less effective pathogen blocking in the *Wolbachia-*infected *A*. *aegypti* line that is currently being used for mosquito control in that country, as well as in other parts of the world. To do so, we orally challenged *Wolbachia*-infected and -uninfected mosquitoes with ZIKV/DENV-1 or ZIKV/DENV-3, and then examined disseminated infection rates and viral load in mosquito heads and thoraces at 7, 14, and 21 days post-infection (dpi). We also examined the effect on virus transmission, using saliva samples collected at 14 and 21dpi.

To gain further insight into the nature of interactions between *Wolbachia*, arboviruses and the mosquito immune system, we examined the effect of *Wolbachia* infection, and ZIKV/DENV co-infection on five candidate genes that had previously been linked to *Wolbachia*-induced viral interference and/or mosquito response to viral infection. We selected two genes that had previously been associated with *Wolbachia* infection and virus interference: Defensin C (DEFC), an antimicrobial peptide that is upregulated during *Wolbachia* infection [47–49], and Transferrin 1 (TSF), an iron-binding protein with a putative immune role, which is upregulated during *Wolbachia* infection [48, 49]. We selected Niemann-Pick Protein C1b (NPC1b), a cholesterol binding protein involved in DENV infection in *A*. *aegypti* [64], due to the known association between *Wolbachia*, viruses and host cholesterol [54, 55]. Finally, we selected two genes that were previously identified as being responsive to viral infection in *A*. *aegypti* but were not characterized in *Wolbachia*-infected mosquitoes: a pupal cuticle protein (PCP) that is downregulated during DENV infection in *A*. *aegypti* [65], and a putative NF-κB repressing factor (NFKBR) that is upregulated during arboviral infection but downregulated by *Wolbachia* [48, 65]. We observed that all of these genes were differentially expressed due to *Wolbachia* infection, ZIKV and/or DENV infection, or *Wolbachia* x virus infection status.

Our results indicated that very few *Wolbachia*-infected mosquitoes become infected by either ZIKV or DENV after experimental co-infection with either ZIKV/DENV-1 or ZIKV/DENV-3, which suggests that *Wolbachia*-induced pathogen interference is effective against multiple pathogens. Our observation that *Wolbachia*-induced inhibition of co-infecting viruses extended to the level of transmission suggests that *Wolbachia*-infected mosquitoes are likely to be a suitable tool for disease control in areas experiencing simultaneous transmission of multiple arboviruses.

## Methods

### Mosquito lines and rearing

The mosquito lines used in this work were as previously described [38]. The *Wolbachia*-infected (Mel) line was originally developed in Australia [28], before being transferred to Brazil, and backcrossed with Brazilian *A*. *aegypti* collected near the residential neighbourhood of Urca, RJ, Brazil. The wildtype, *Wolbachia*-uninfected line (WT) used in these experiments was originally collected from the same area in 2016. Mosquito eggs were regularly collected from this site, and WT adult males were introduced into both the Mel and WT colonies each generation in order to maintain similar genetic backgrounds between the lines. All mosquitoes were kept in a climate-controlled insectary, under previously described conditions [56].

### Virus culture and titration

The ZIKV strain used in these experiments (ZIKV/*H*. *sapiens*/Brazil/BRPE243/2015) was isolated in 2015 [38, 66]. The DENV-1 strain (DENV-1/*H*. *sapiens*/Brazil/Contagem/MG/BRMV09/2015) was isolated from human blood in Contagem, MG, Brazil during 2015 [42]. The DENV-3 strain (DENV-3 MG20 (375)) was isolated from human blood in Brazil during 2012 [49]. Virus stocks were maintained at 28°C, in C6/36 cells in Leibowitz L-15 medium supplemented with 10% fetal bovine serum (FBS), 1% penicillin/streptomycin (Gibco), as previously described [38, 67]. Fresh supernatant from infected C6/36 cells was harvested 6–7 days after infection. The RNA from 50μL of fresh infected supernatant was extracted in 50μL of squash buffer (1mM EDTA, 50mM NaCl, 10mM Tris, pH 8.2) and 0.6μL of proteinase K (Qiagen), and then quantified via RT-qPCR using the LightCycler Multiplex RNA virus Master (Roche). The quantification protocol is described in more detail below. Based on these results, viruses were diluted in fresh, sterile media to approximately equal titres (Table 1), and these were used in mosquito oral infections, without ever being frozen. In each experiment, the virus with the lowest titre, which was always DENV-1, was used as a reference to calculate the dilutions of the other viruses.

**Table 1:** Viral titres.

	Initial RT-qPCR estimate of titre of infected supernatant (copies/mL)	Dilution factor used in blood meal	Plaque assay titration of undiluted virus-infected supernatant (PFU/mL)
**Experiment 1**			
ZIKV	6.2x10^10^	282x	8.2x10^6^
DENV-1	1.7x10^8^	NA	2.5x10^6^
DENV-3	1.9x10^8^	1.14x	6.0x10^4^
**Experiment 2**			
ZIKV	6.8x10^11^	220x	3.5x10^8^
DENV-1	3.1x10^9^	NA	3.5x10^7^
DENV-3	6.7x10^11^	218x	4.5x10^6^

Notes: Mono-infection experiments were performed with viruses from Experiment 2. Dilution factors were used to feed mosquitoes with approximately equivalent viral titres, based on the RT-qPCR estimates of viral titre. NA: Virus was not diluted when fed to mosquitoes.

Undiluted, virus-infected supernatant was also used to perform plaque assays as an additional method of quantification. Plaque assays for DENV-1 and DENV-3 were performed using BHK 21 cells, while plaque assays for ZIKV were performed using VERO cells. Briefly: 4 x 10^5^ cells were added to each well in a 6-well plate (Kasvi) containing DMEM media (Gibco) supplemented with 5% inactivated FBS (Gibco), and then incubated overnight at 37°C, with 5% CO_2_. Then, infected supernatant was serially diluted from 10^−3^ through 10^−7^ and 400µL added to the plate. After a one-hour incubation at 37°C, the media was removed and 4mL of DMEM containing 1.5% carboxymethylcellulose (Synth) and 2% FBS was added to each well. The cells were then incubated at 37°C for 6 days in an incubator with 5% CO_2_. After this period, the cells were fixed with 3.7% formaldehyde (Exodo Cientifica) for 1 hour, and then stained with 1% crystal violet (Merck) for 20 minutes. Plaque numbers were counted to determine the number of plaque forming units per millilitre of virus [68, 69].

### Experimental infection and sample collection

Four to six day-old adult female mosquitoes were starved overnight prior to experimental infection. For co-infection meals, 1mL of each virus culture was mixed with 1mL of fresh human blood. For mono-infected meals, 2mL of virus was mixed with 1mL of fresh human blood. For mock-infected meals, 1mL of fresh human blood was mixed with 2mL of supernatant collected from uninfected C6/36 cells. Feeding was performed using a membrane feeding system with a circulating water bath to keep temperatures at 37°C. Immediately post-feeding, mosquitoes were knocked down with carbon dioxide, and sorted on ice. All females that were not fully engorged were discarded. The remainder were returned to cages and maintained on 10% sucrose, which was changed daily. Mosquitoes were collected on ice at 7, 14 and 21 days post-infection, and then stored at -80°C until the time of processing. One mono-infection assay was performed for ZIKV, DENV-1 and DENV-3. Two ZIKV/DENV-1 co-infection assays, and two ZIKV/DENV-3 assays were performed. Samples were dissected on dry ice using microscissors to remove the head/thorax (minus legs and wings) from the remainder of the carcass, and then returned to storage at -80°C.

### Saliva collection and injection

Saliva was collected from Mel and WT mosquitoes during one ZIKV/DENV-3 co-infection experiment at both 14 and 21dpi. Mosquitoes were starved overnight to promote feeding. The next day, mosquitoes were knocked down with carbon dioxide and kept on ice while legs and wings were removed. Mosquitoes were allowed to revive, and then saliva was collected by inserting the proboscis into a sterile pipet tip containing 5μL of a 1:1 solution of sterile fetal bovine serum and 30% sucrose. Mosquitoes were then permitted 30 minutes to salivate. Saliva from mosquitoes, that were still alive after the 30 minutes, was collected in sterile tubes and stored at -80°C [38, 42].

Using a Nanoject II (Drummond), saliva samples were intrathoracically injected into 4–6 day-old WT mosquitoes that had not previously been challenged by ZIKV or DENV, in order to determine if they contained infectious virus. A total of 32 saliva samples (WT 14dpi– 7, Mel 14dpi– 7, WT 21dpi– 9, Mel 21dpi—9) were injected into 435 WT females. A fresh glass needle was used for each saliva sample. Between 9 and 16 mosquitoes were injected per saliva, with each mosquito injected with 207nL of saliva. Injected mosquitoes were maintained on 10% sucrose for 5 days, with the sucrose changed daily. Including those mosquitoes that died as a result of injection-induced trauma, there was a mean survival rate of 84.65% for the experiment. The presence or absence of ZIKV and DENV was analysed for a maximum of 8 injected mosquitoes per saliva, corresponding to an average sampling rate of 58.12% across all samples, and a total of 251 injected mosquitoes sampled. These samples were stored at -80°C, and whole mosquito samples were processed.

### Quantification of ZIKV and DENV levels

RNA was extracted from mosquito samples using the High Pure Viral Nucleic Acid Kit (Roche) according to the manufacturer’s instructions. Samples were diluted to 50ng/μL and then levels of ZIKV and DENV were quantified by RT-qPCR using the LightCycler Multiplex RNA virus Master (Roche) and a LightCycler 96 (Roche). All assays were run in multiplex for ZIKV, DENV and *RpS17*, which served as a host control in each reaction. ZIKV primers and probe were as previously described (ZIKV 835; ZIKV911c –ZIKV 860-FAM) [70]. DENV primers and probe functioned for both DENV-1 and DENV-3 (S1 File). Thermocycling conditions were as follows: an initial reverse transcription step at 50°C for 5 min; RT inactivation/initial denaturation at 95°C for 20 sec, and 40 cycles of 95°C for 3 sec then 60°C for 30 sec. The total reaction volume was 10μL (LightCycler Multiplex RNA virus Master (Roche), 0.5μL of 10μM primers and 0.1μL of 10μM probe, and 125ng of RNA template). Each sample was run in duplicate. Viral load was determined by absolute quantification by comparison with serial dilution of the amplicon for each gene, cloned and then amplified in the pGEMT-Easy plasmid (Promega) [38, 41]. Each plate included positive and negative controls for each virus. A detection threshold was set at 100 copies of each virus, as this dilution did not reliably amplify for either the DENV or ZIKV standard curves. Samples below this threshold were considered to have no detectable virus. Final viral load data were calculated as the total number of copies per whole head/thorax sample.

### Gene expression

Samples for the gene expression assay were collected at 24 hours post-infection during one of the ZIKV/DENV-1 co-infection assays. The assay included mock infection treatments for the Mel and WT lines. The assay also included DENV-1 and ZIKV mono-infection treatments, and a ZIKV/DENV-1 co-infection treatment, with these treatments infected exactly as described above. Samples were collected on ice and stored at -80°C. Total RNA was extracted from whole mosquitoes using the standard Trizol protocol (ThermoFisher). First strand cDNA synthesis was conducted using M-MLV reverse transcriptase (Promega), as previously described [49]. Expression levels of Defensin C (Vectorbase ID: AAEL003832-RA, [71]), NF-κB repressing factor, putative (AAEL008415-RB), Niemann-Pick Protein C1b (AAEL009531-RA), pupal cuticle protein, putative (AAEL022261-RA), and transferrin 1 (AAEL015458-RA) were quantified relative to the *A*. *aegypti* ribosomal protein s17 (*RpS17*, AAEL004175-RA) using SYBR green (ThermoFisher), and a LightCycler® 96 (Roche). Primers for these genes were either designed using Primer 3 V0.4.0 (http://bioinfo.ut.ee/primer3-0.4.0/), (S1 File) or were as previously described [49, 64, 65]. Quantification for each sample was performed in duplicate relative to levels of *RpS17*. Each reaction contained the following: 2μL cDNA sample (at 1:10 dilution), 5μL SYBR Green PCR Master Mix (ThermoFisher), 0.5μL of each primer (10mM), 2 μL DNAse/RNAse-free water). The RT-qPCR run profile was as follows: 95°C for 10 mins, 40 cycles of 95°C for 15s then 60°C for 30s, followed by a melt curve from 60°C to 95°C. Mean normalized expression values were calculated using Q-Gene [72].

### Statistical analysis

Prevalence data for the mono-infection experiments, or other comparisons where there were two outcomes, were compared using Fisher’s exact test. Prevalence data for co-infection experiments, which involved four potential infection outcomes, were compared using Chi-square tests. Viral load data included only samples that were positive for viral infection. These data were assayed for normality using the D’Agostino & Pearson omnibus normality test. These data were non-parametrically distributed and were therefore compared using Mann-Whitney U tests. ZIKV: DENV ratios for co-infected mosquitoes were LOG_10_ transformed. As these data were normally distributed, they were compared across time points using a standard One-way ANOVA, then pairwise comparisons were performed using Tukey’s multiple comparison test. Gene expression data were compared using a generalized linear model of regression for each gene, with expression as the test variable, and *Wolbachia* infection status, infecting virus, and *Wolbachia* x viral infection as the explanatory variables. Pairwise comparisons were made for these data using Mann-Whitney U tests. For analyses where more than 10 Mann-Whitney U tests were performed, a 5% false discovery rate was applied as a multiple test correction. All statistical analyses were performed using Prism 6.0h (Graphpad), with the exception of the generalized linear models, which were performed using SPSS 17.0 (IBM).

### Ethics statement

Prior written consent was obtained from the willing, adult volunteer from whom the blood was drawn. The blood was drawn by a trained medical professional in accordance with existing institutional guidelines, and the use of human blood in these experiments was approved by The Committee for Ethics in Research (CEP)/ FIOCRUZ (License—CEP 732.621). These procedures were conducted according to Brazilian laws 196/1996 and 01/1988, which govern human ethics issues in scientific research in compliance with the National Council of Ethics in Research (CONEP).

## Results

### *Wolbachia* inhibits infection with ZIKV, DENV-1 and DENV-3 in mono-infection

In this study we sought to determine if the pathogen blocking properties of *Wolbachia* infection could still function effectively if *Wolbachia*-infected mosquitoes were challenged with two viruses simultaneously. First, we examined the effects of *Wolbachia* infection in *A*. *aegypti* individually on the three viruses we intended to use in co-infection assays. These viruses (ZIKV BRPE strain, DENV-1 and DENV-3) were all isolated in Brazil. We had previously determined that *w*Mel infection in *A*. *aegypti* caused pathogen blocking against both ZIKV and DENV-3 [38, 49], while this isolate of DENV-1 had not previously been examined with *Wolbachia*-infected mosquitoes. We used RT-qPCR with virus-specific TaqMan probes to quantify ZIKV or DENV load in *w*Mel-infected (Mel) and wildtype (WT) mosquito head/thorax samples, in order to assess disseminated infection, at 7, 14 and 21 days post-oral infection (dpi). Data are provided in S2 File.

**Fig 1:**
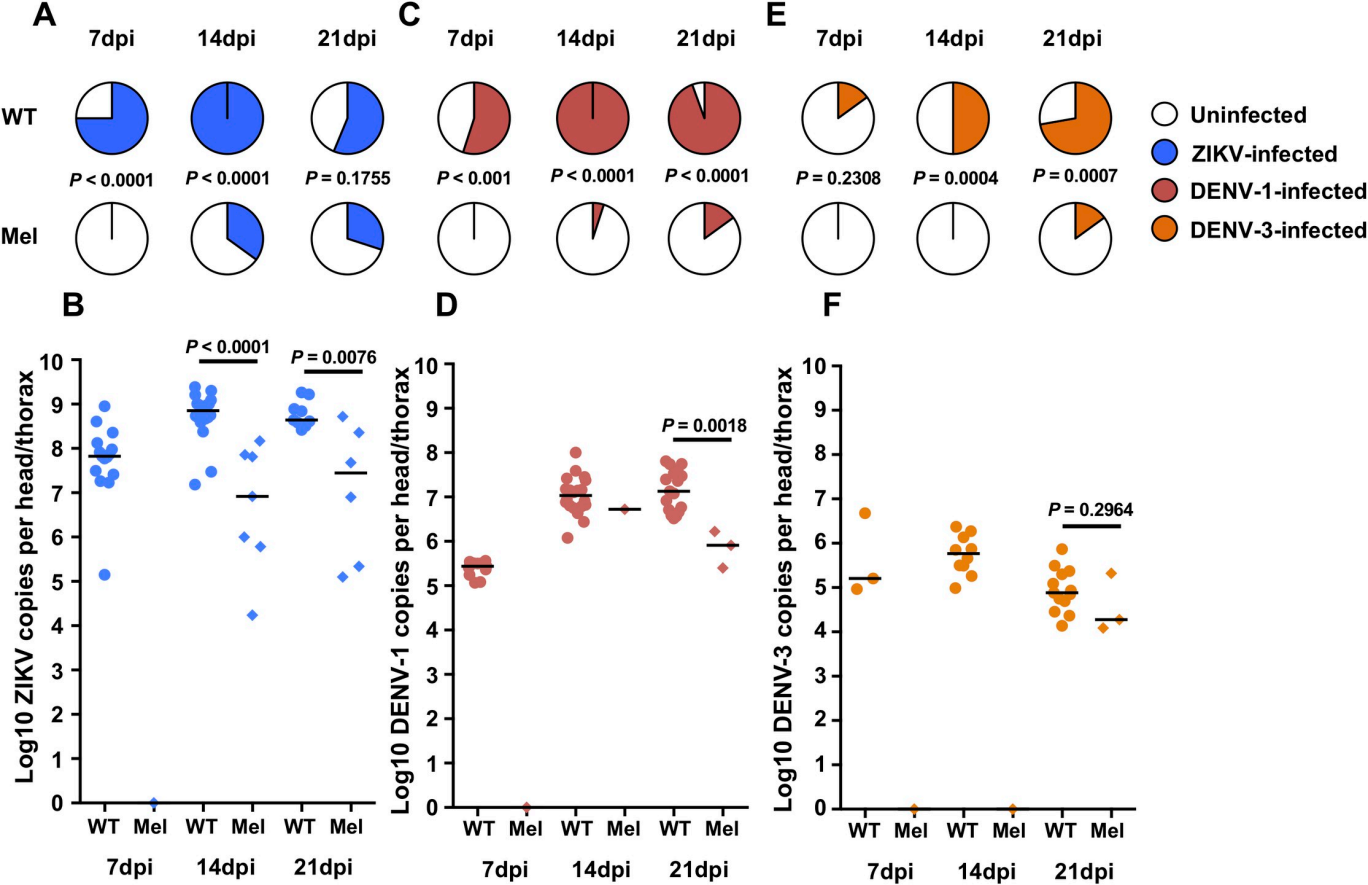
*Wolbachia* inhibits infection with ZIKV, DENV-1 and DENV-3 isolates during mono-infection. Wildtype (WT, circles) and *w*Mel-infected (Mel, diamonds) *A*. *aegypti* were orally challenged with ZIKV (**A**,**B**, blue), DENV-1 (**C**,**D**, red), or DENV-3 (**E**,**F**, orange), individually. Mosquitoes were collected at 7, 14 and 21 days post-infection, and disseminated infection levels were determined for individual head/thorax samples via RT-qPCR using TaqMan probes. Mel mosquitoes had significantly lower prevalence of infection for all viruses, at all time points with the exception of DENV-3 at 7dpi. Pie charts depict prevalence of infection, with *P* values determined via Fisher’s exact test. Dot plots depict viral load for infected samples only, with *P* values determined via Mann-Whitney *U* test for all comparisons where there were sufficient numbers of virus-infected Mel samples. Black bars in the dot plots represent treatment medians. Each dot represents a single mosquito sample. *N* = 16–20 samples per treatment.

For ZIKV, prevalence of infection was significantly lower amongst Mel mosquitoes at 7 (Fisher’s exact test; *P* < 0.0001) and 14dpi (Fisher’s exact test; *P* < 0.0001) (Fig 1A). Prevalence at 21dpi was 56.3% in WT mosquitoes, and 30% in Mel mosquitoes, however this difference was not statistically significant (Fisher’s exact test; *P* = 0.1755). In terms of viral load (Fig 1B), no Mel mosquito samples were infected at 7dpi (Mann-Whitney U; *U* = 6, *P* < 0.0001), while at 14dpi and 21dpi, ZIKV load in the infected samples was significantly lower in Mel mosquitoes than WT mosquitoes (Mann-Whitney U; 14dpi–*U* = 6, *P* < 0.001; 21dpi—*U* = 5, *P* = 0.0076). Median ZIKV load in infected WT samples was approximately 10^9^ copies at both 14 and 21dpi.

For DENV-1, prevalence was significantly lower for Mel mosquitoes at all three time points examined (Fisher’s exact test; 7dpi–*P* = 0.001; 14dpi–*P* < 0.0001; 21dpi—*P* < 0.0001). No Mel samples displayed infection at 7dpi (Fig 1C), while WT infection levels were greater than 90% at both 14 and 21dpi. As only 1 of 20 Mel samples became infected at 14dpi, statistical comparison of viral load was not possible (Fig 1D). At 21dpi, viral load in the three infected Mel samples was significantly lower than for WT mosquitoes (Mann-Whitney U; *U* = 0, *P* = 0.0018). Median DENV-1 load in WT samples was approximately 10^7^ copies at both 14 and 21dpi.

For DENV-3, at 7dpi, prevalence of infection amongst WT mosquitoes was low, and not significantly different to the Mel treatment, where no infection was observed (Fisher’s exact test; *P* = 0.2308). However, prevalence in Mel mosquitoes was significantly lower than in WT at both 14dpi (Fisher’s exact test; *P* = 0.0004) and 21dpi (Fisher’s exact test; *P* = 0.0007) (Fig 1E). Due to low prevalence in Mel samples, DENV-3 load could not be compared between WT and Mel mosquitoes at 7 or 14dpi (Fig 1F). At 21dpi, only 3 of 20 Mel samples were infected and viral load was not significantly different to that of WT mosquitoes (Mann-Whitney U; *U* = 11, *P* = 0.2964). Median DENV-3 load in WT samples was approximately 10^6^ copies at 14dpi and approximately 10^5^ copies at 21dpi.

### Pathogen blocking is not affected by ZIKV/DENV co-infection in *Wolbachia*-infected mosquitoes

We examined the effects of *Wolbachia* on ZIKV/DENV co-infection in WT and Mel mosquitoes by performing co-oral infection assays where viruses were fed to mosquitoes in approximately equivalent titres (as determined by RT-qPCR in the hours prior to infection). Two experimental infections were performed for ZIKV/DENV-1, and two for ZIKV/DENV-3, all using fresh virus. Head/thorax samples were then assayed for the presence of both viruses using a multiplex RT-qPCR assay. Raw data are provided in S2 File.

When examining prevalence of viral infection for these experiments, we classified the samples as uninfected (no detectable infection with either virus), DENV-infected (only DENV detected), ZIKV-infected (only ZIKV detected), or co-infected (both viruses detected). These data were compared by Chi-square test. Viral load data from both replicate experiments were examined as one data set, however data were examined independently for each virus.

**Fig 2:**
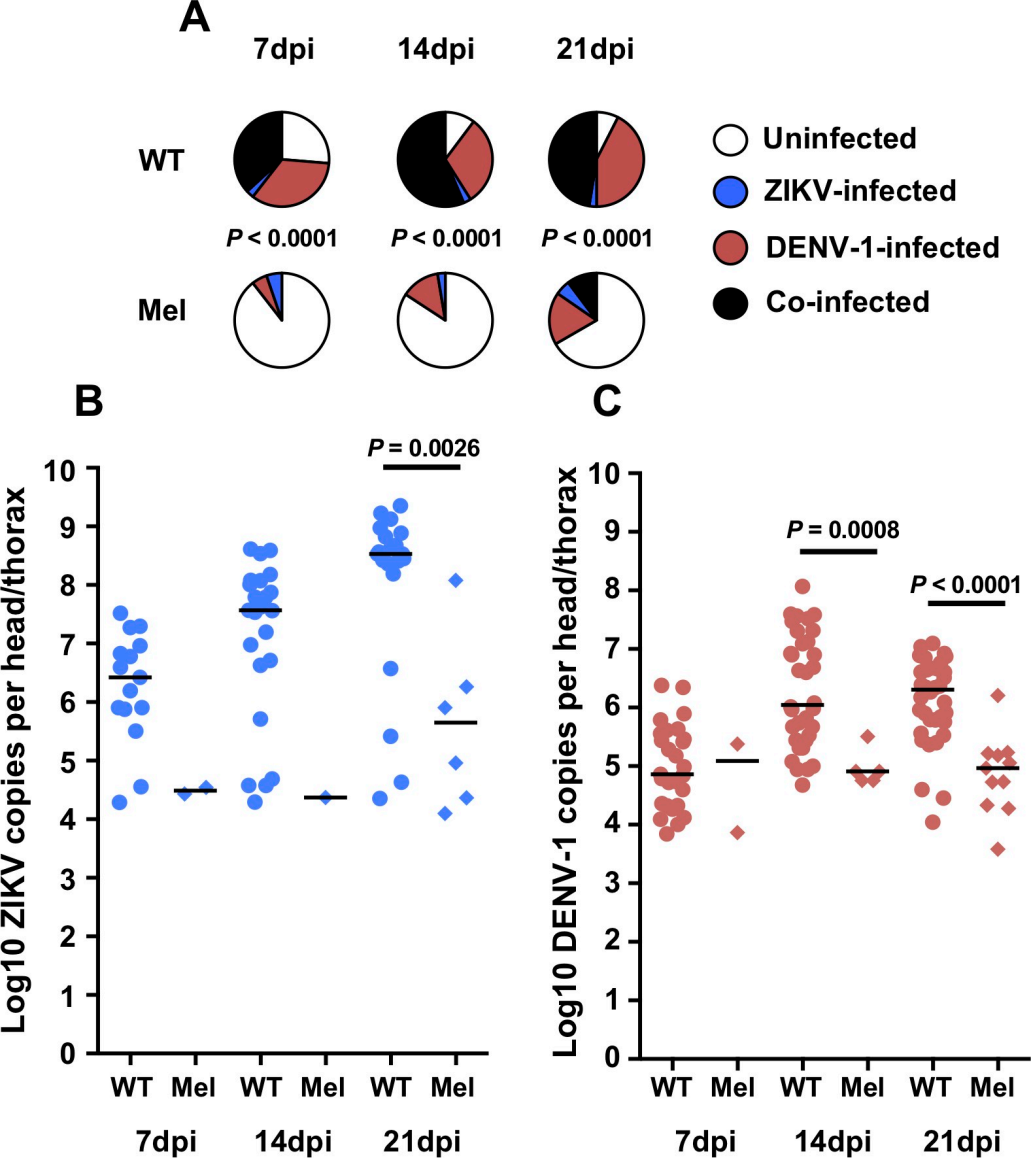
*Wolbachia* inhibits infection with ZIKV and DENV-1 during co-infection. Wildtype (WT, circles) and *w*Mel-infected (Mel, diamonds) *A*. *aegypti* were orally challenged with both ZIKV (blue) and DENV-1 (red), with the viruses offered at approximately equivalent titres. Mosquitoes were collected at 7, 14 and 21 days post-infection, and disseminated infection levels were determined for individual head/thorax samples via RT-qPCR using specific TaqMan probes. Mel mosquitoes displayed significantly lower prevalence of infection than WT at all three time points **(A)**. Mel mosquitoes had significantly lower ZIKV **(B)** and DENV-1 **(C)** load than WT mosquitoes. Data represent two replicate experiments. Pie charts depict prevalence of infection, with *P* values determined via Chi-square test. Dot plots depict viral load for infected samples only, with *P* values determined via Mann-Whitney *U* test for all comparisons where there were sufficient numbers of virus-infected Mel samples. Black bars in the dot plots represent treatment medians. Each dot represents a single mosquito sample. *N* = 38–40 samples per treatment.

For the ZIKV/DENV-1 co-infection experiments, prevalence of infection was significantly lower for Mel mosquitoes than WT mosquitoes at all three time points (Chi-square test; 7dpi–*X*^*2*^ = 35.49, *df* = 3, *P* < 0.0001; 14dpi–*X*^*2*^ = 46.66, *df* = 3, *P* < 0.0001; 21dpi–*X*^*2*^ = 32.52, *df* = 3, *P* < 0.0001) (Fig 2A). The overall prevalence of infection in Mel mosquitoes increased over time, and reached 33.33% at 21dpi, however only 4/39 samples (10.25%) had detectable co-infection at this time point. For WT mosquitoes, few samples were infected solely by ZIKV, with most samples positive only for DENV-1, or for both viruses.

Due to low infection rates in Mel mosquitoes, statistical comparison of ZIKV load between Mel and WT samples from these experiments was possible only at 21dpi, where Mel ZIKV load was significantly lower than for WT mosquitoes (Mann-Whitney U test; *U* = 13, *P* = 0.0026) (Fig 2B). DENV-1 load was significantly lower in Mel mosquitoes than WT at both 14dpi (Mann-Whitney U test; *U* = 13, *P* = 0.0008) and 21dpi (Mann-Whitney U test; *U* = 40, *P* < 0.0001) (Fig 2C).

**Fig 3:**
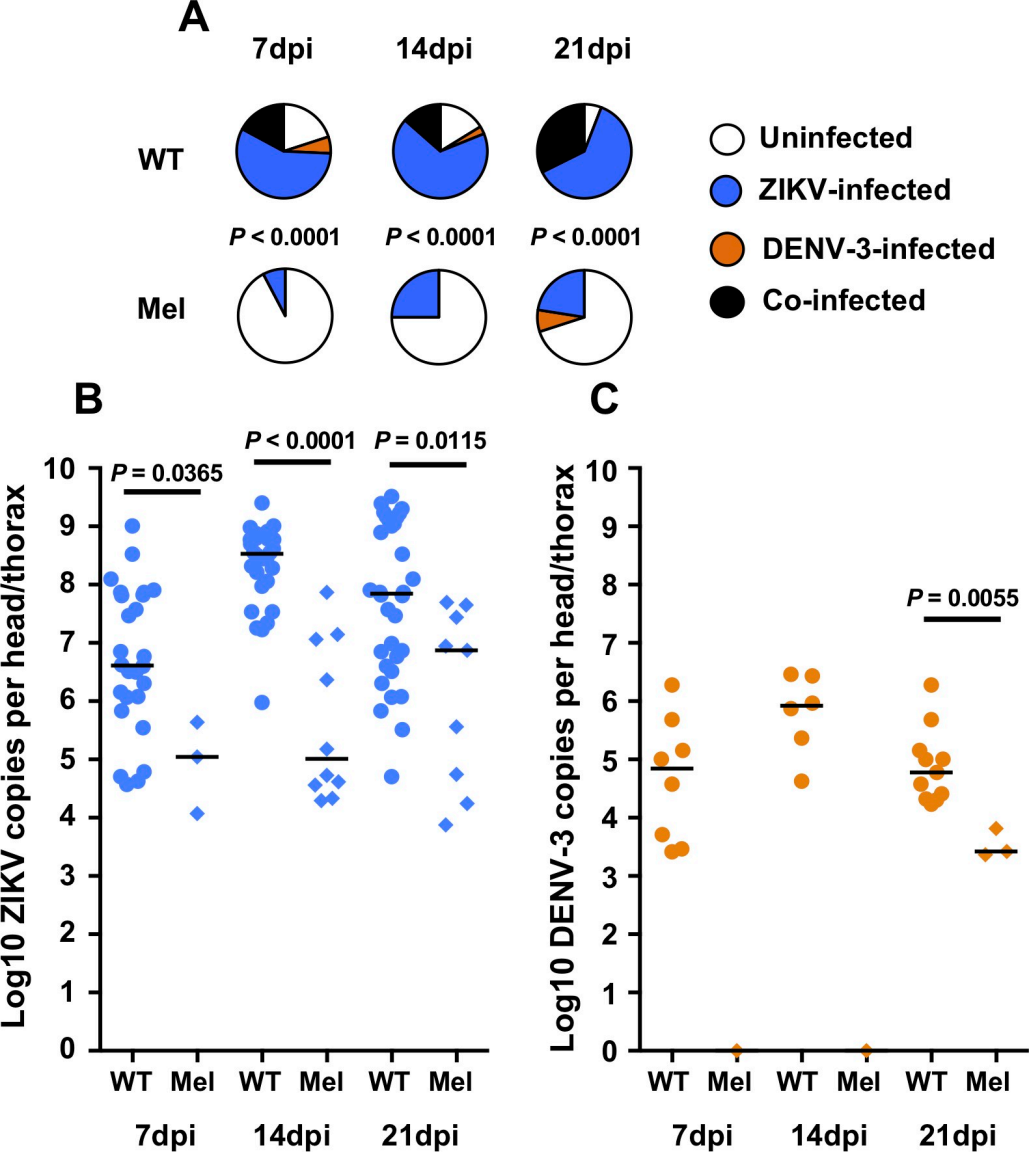
*Wolbachia* inhibits infection with ZIKV and DENV-3 during co-infection. Wildtype (WT, circles) and *w*Mel-infected (Mel, diamonds) *A*. *aegypti* were orally challenged with both ZIKV (blue) and DENV-3 (orange), with the viruses offered at approximately equivalent titres. Mosquitoes were collected at 7, 14 and 21 days post-infection, and disseminated infection levels were determined for individual head/thorax samples via RT-qPCR using specific TaqMan probes. Mel mosquitoes displayed significantly higher proportion of uninfected mosquitoes than WT at all three time points **(A)**. Mel mosquitoes had significantly lower ZIKV **(B)** and DENV-3 **(C)** load than WT mosquitoes. Data represent two replicate experiments. Pie charts depict prevalence of infection, with *P* values determined via Chi-square test. Dot plots depict viral load for infected samples only, with *P* values determined via Mann-Whitney *U* test for all comparisons where there were sufficient numbers of virus-infected Mel samples. Black bars in the dot plots represent treatment medians. Each dot represents a single mosquito sample. *N* = 34–40 samples per treatment.

For the ZIKV/DENV-3 co-infection experiments, prevalence of infection was again significantly lower for Mel mosquitoes than WT mosquitoes at all three time points (Chi-square test; 7dpi–*X*^*2*^ = 40.02, *df* = 3, *P* < 0.0001; 14dpi–*X*^*2*^ = 28.35, *df* = 3, *P* < 0.0001; 21dpi–*X*^*2*^ = 41.12, *df* = 3, *P* < 0.0001) (Fig 3A). As for the ZIKV/DENV-1 experiments the overall prevalence of infection in Mel mosquitoes increased slightly over time, reaching 30% at 21dpi. We observed no co-infected Mel mosquitoes in either ZIKV/DENV-3 experiment. In contrast to the ZIKV/DENV-1 co-infection experiments where DENV-1 infection was more prevalent than ZIKV, most WT mosquitoes were infected by ZIKV alone or by both viruses, with few infected only by DENV-3.

In ZIKV/DENV-3 co-infection experiments, ZIKV load was significantly lower in Mel samples than in WT samples at all three time points (Mann-Whitney U test; 7dpi–*U* = 12, *P* = 0.0365; 14dpi–*U* = 9, *P* < 0.0001; 21dpi–*U* = 65, *P* = 0.0115) (Fig 3B). Comparison of DENV-3 load was only possible at 21dpi, as all Mel samples were uninfected at the other time points (7dpi– 0/39; 14dpi– 0/40). We again observed significantly lower DENV-3 load in Mel mosquitoes (Mann-Whitney U test; *U* = 0, *P* = 0.0055) (Fig 3C).

To determine whether co-infection affected pathogen blocking in Mel mosquitoes, we compared the prevalence and viral load of each virus when fed singly or in co-infection. We observed no significant difference in the prevalence of ZIKV infection when fed in mono- or in co-infection with DENV-3. Likewise, there was no difference in prevalence of either DENV strains fed in mono-infection or co-infection with ZIKV. The one exception to this was with ZIKV/DENV-1 co-infection at 14dpi, where prevalence of infection was actually lower than for ZIKV mono-infection (Fisher’s exact test; *P* = 0.0014). However, there was no significant difference observed between these treatments at either 7dpi or 21dpi.

For viral load, we observed no significant difference between Mono- or co-infected Mel mosquitoes for any virus for all but one comparison. This occurred for DENV-1 levels in mono-infected Mel mosquitoes compared to those challenged with ZIKV/DENV-1, where we observed significantly lower DENV-1 load associated with co-infection (Mann-Whitney U test; *U* = 2, *P* = 0.0220), although there were only three infected samples in the mono-infection condition.

### Co-infection alters infection dynamics of ZIKV and DENV in wildtype mosquitoes

We sought to assess changes to the dynamics of DENV and ZIKV infection in co- and mono-infected WT mosquito head/thoraces. We observed that the prevalence of ZIKV infection was significantly decreased during co-infection with DENV-1 at 7dpi (Fisher’s exact test; *P* = 0.0135) and 14dpi (Fisher’s exact test; *P* = 0.0004). The prevalence of ZIKV was also decreased during co-infection with DENV-3 at 14dpi (Fisher’s exact test; *P* = 0.0447), but increased with co-infection with DENV-3 at 21dpi (Fisher’s exact test; *P* = 0.0027). Additionally, the prevalence of DENV-3 infection was significantly decreased during co-infection with ZIKV at 14dpi and 21dpi (Fisher’s exact test; 14dpi—*P* = 0.0123, 21dpi—*P* = 0.088). However, the prevalence of DENV-1 infection was unaffected by co-infection with ZIKV.

We observed a similar effect for ZIKV load during co-infection, with ZIKV load during co-infection with DENV-1 significantly decreased at 7dpi (Mann-Whitney U test; *U* = 21, *P* < 0.0001) and 14dpi (Mann-Whitney U test; *U* = 34, *P* < 0.0001), compared to ZIKV mono-infection. A similar decrease in ZIKV load was observed during co-infection with DENV-3 (Mann-Whitney U test; 7dpi–*U* = 99, *P* = 0.0086; 14dpi–*U* = 170, *P* = 0.0093). We also observed significantly decreased DENV-1 levels during co-infection with ZIKV at both 14dpi and 21dpi (Mann-Whitney U test; 14dpi–*U* = 194, *P* = 0.0082; 21dpi–*U* = 65, *P* < 0.0001). However, there was no effect of co-infection on DENV-3 levels.

### Viral load in ZIKV/DENV co-infected mosquitoes is dominated by ZIKV

Analysis of our prevalence data in the co-infection assays suggested that more WT samples were positive for DENV-1 than for ZIKV during ZIKV/DENV-1 co-feeding, while by comparison more WT samples were positive for ZIKV than for DENV-3 during ZIKV/DENV-3 co-feeding. To see if a similar trend occurred for viral load in co-infected samples, we examined the ratio of ZIKV copies to DENV copies, across all co-infection assays. These comparisons were only made for WT mosquitoes, as there were too few co-infected Mel mosquitoes to perform statistical analysis.

**Fig 4:**
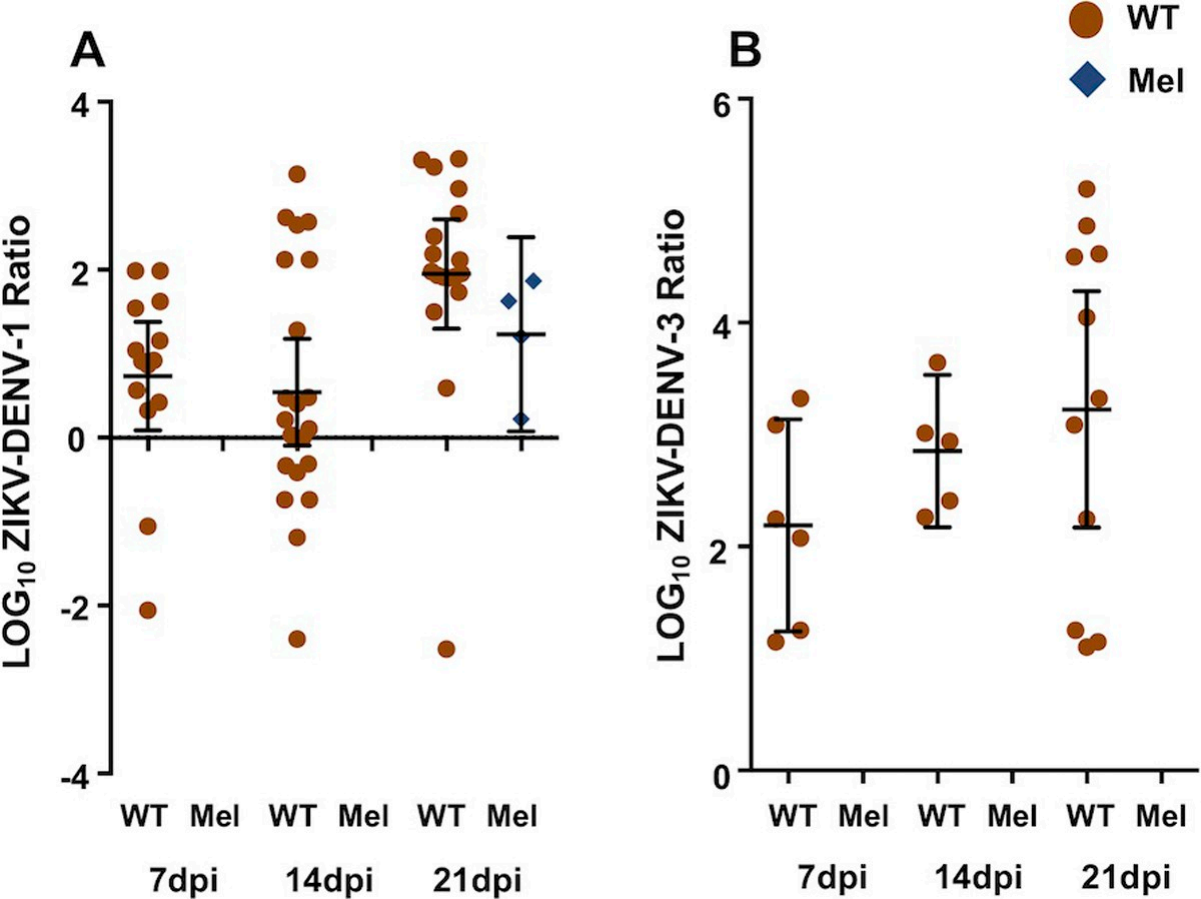
Co-infected *A*. *aegypti* generally had higher levels of ZIKV than either DENV-1 or DENV-3. Graphs depict the LOG_10_ ZIKV-DENV-1 ratio **(A)** and the LOG_10_ ZIKV-DENV-3 ratio **(B)** for all WT (brown) and Mel (blue) samples found to be positive for infection with both viruses via RT-qPCR. Ratios greater than zero indicate ZIKV levels were greater than DENV levels, for that samples, while ratios less than zero indicate the opposite. Data were compiled from two ZIKV/DENV-1 and two ZIKV/DENV-3 co-infection experiments. Black lines represent mean ZIKV:DENV ratio and the 95% confidence interval estimate of the mean ratio.

For the ZIKV/DENV-1 co-infection assays (Fig 4A), the majority of co-infected samples had higher levels of ZIKV than DENV-1 (LOG_10_ Ratio > 0). Comparison of LOG_10_ ZIKV:DENV over the course of infection revealed that this ratio changed with time (One-Way ANOVA; *F* = 4.339, *P* = 0.0082). This effect was due to differences between the samples at 14dpi and 21dpi, with the latter time point having more samples with higher levels of ZIKV than DENV-1 (Mann-Whitney U test; *U* = 10, *P* = 0.0257). For the ZIKV/DENV-3 co-infection assays (Fig 4B), all samples had higher levels of ZIKV than DENV-3, and statistical comparison revealed that there was no significant change to ZIKV:DENV ratio over time (One-Way ANOVA; *F* = 1.318, *P* = 0.2010). Across all experiments, there were four co-infected Mel samples. All of these samples were from mosquitoes collected at 21 days post-infection with ZIKV/DENV-1, and all four had higher levels of ZIKV than DENV-1.

### *Wolbachia* blocks viral transmission during co-infection

**Fig 5:**
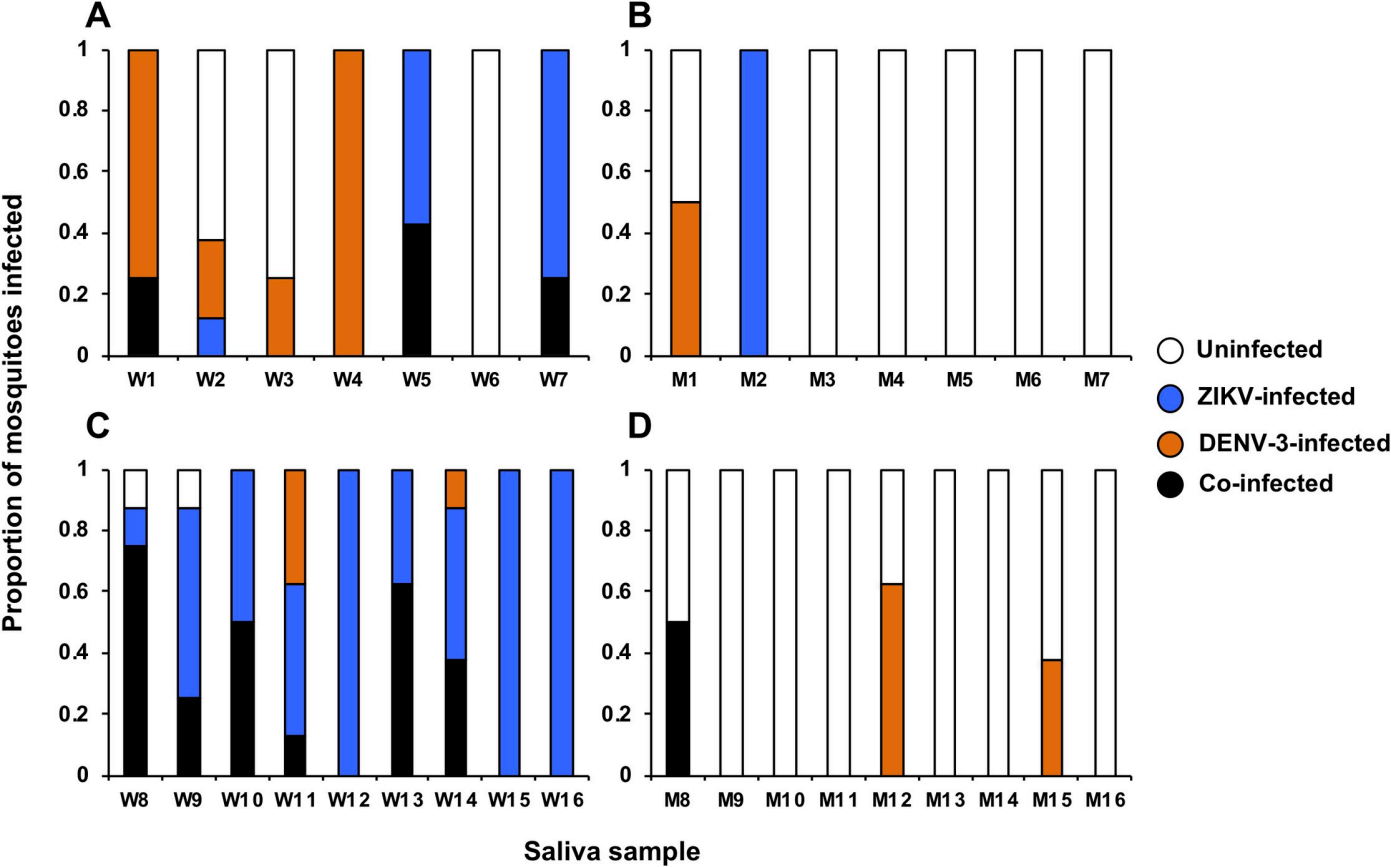
Co-infection with ZIKV and DENV-3 does not increase virus prevalence in the saliva of *Wolbachia*-infected mosquitoes. Saliva was collected from individual ZIKV/DENV-3-challenged WT and Mel mosquitoes at 14 and 21dpi. These saliva samples were then injected into naïve WT mosquitoes and presence/absence of ZIKV and DENV-3 determined 5 days later via RT-qPCR. Each bar represents a cohort of mosquitoes injected with a single saliva sample collected from WT mosquitoes (W) at 14dpi **(A)**, Mel mosquitoes (M) at 14dpi **(B)**, WT mosquitoes at 21dpi **(C)**, and Mel mosquitoes at 21dpi **(D)**. Overall prevalence of infection was significantly lower for Mel mosquitoes at both time points (Fisher’s exact test; *P* < 0.0001).

We collected saliva samples from WT and Mel mosquitoes at 14dpi and 21dpi during one of the ZIKV/DENV-3 co-infection experiments in order to examine the effect of co-infection on the ability of *Wolbachia* to restrict virus transmission in mosquitoes. Raw data are provided in S3 File. Sixteen saliva from WT mosquitoes and 16 from Mel mosquitoes were injected into 435 female WT mosquitoes (an average of 13.59 mosquitoes per saliva sample) that were not previously challenged with ZIKV or DENV. A total of 251 mosquitoes, an average of 58.12% of the mosquitoes from each group, were assayed for ZIKV and DENV infection using RT-qPCR. Mel saliva selection was intentionally biased to include the saliva collected from all of the Mel mosquitoes where the head/thorax samples were positive for at least one virus. WT saliva samples were selected randomly. Saliva samples that produced at least one subsequent infection in the injected WT mosquitoes were classified as infectious. Significantly more WT saliva samples (15/16–93.75%) than Mel (5/16–31.25%) were infectious (Fisher’s exact test; *P* < 0.0001). At 14dpi, 6/7 (85.71%) WT saliva (Fig 5A) and 2/7 (28.57%) Mel saliva (Fig 5B) were infectious, and WT saliva generated significantly more subsequent infections (Fisher’s exact test; *P* < 0.0001). While at 21dpi, 9/9 (100%) WT saliva (Fig 5C) and 3/9 (33.33%) Mel saliva (Fig 5D) were infectious, and WT saliva again generated significantly more subsequent infections (Fisher’s exact test; *P* < 0.0001).

### *Wolbachia* and virus infection alter mosquito gene expression

We examined whether *Wolbachia* infection altered the expression of key genes at 24 hours post-infection with ZIKV, DENV-1 or ZIKV/DENV-1 co-infection, in comparison to Mock-infected mosquitoes. We compared the expression levels of 5 genes associated with viral infection and/or *Wolbachia* infection in *A*. *aegypti* via RT-qPCR across 10–15 mosquitoes per treatment. Raw data and statistical output are provided in S4 File. The expression data were compared using a generalized linear model (GLM) of regression for each gene, and then were compared pairwise using Mann-Whitney U tests.

**Fig 6:**
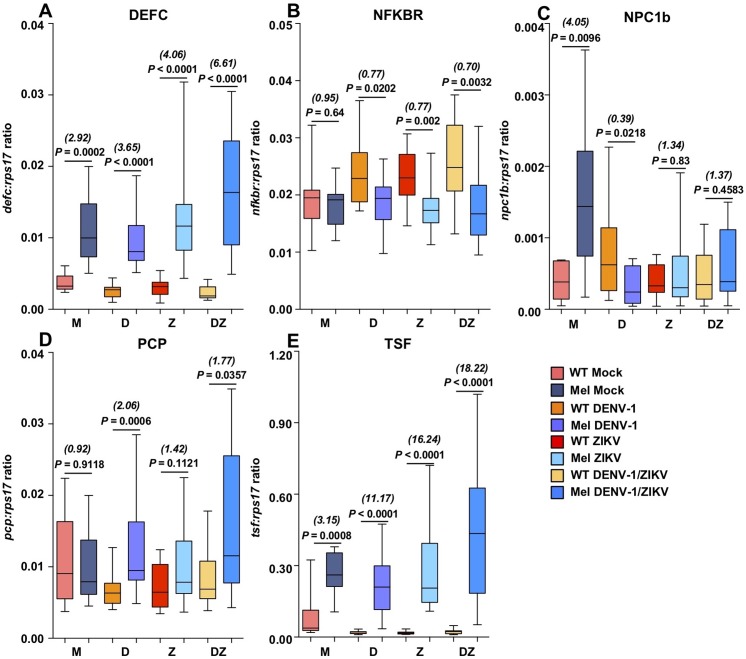
Key infection response genes are differentially affected by infection with *Wolbachia*, DENV-1 and ZIKV. Box and whisker plots depicting expression levels of key genes *Defensin C* (DEFC) **(A)**, *NF-κB repressing factor*, *putative* (NFKBR) **(B)**, *Niemann-Pick Protein C1b* (NPC1b) **(C)**, *pupal cuticle protein*, *putative* (PCP) **(D)**, and *transferrin 1* (TSF) **(E)** for WT and Mel mosquitoes at 1 day post-infection with a mock blood meal (M) DENV-1 (D) ZIKV (Z), ZIKV/DENV-1 (DZ), as determined via RT-qPCR. Mean normalized expression values were calculated relative to *RpS17*, and compared using generalized linear models, and pairwise via Mann-Whitney U tests. *P* values presented in the figure represent Mann-Whitney U tests. Numbers in italics represent average fold change for Mel mosquitoes relative to WT mosquitoes in each treatment.

Expression levels of the antimicrobial peptide Defensin C (DEFC, AAEL003832-RA) were significantly elevated in Mel mosquitoes under all conditions (GLM; *W* = 115.896, *df* = 3, *P* < 0.0001) (Fig 6A), with significant changes in expression also occurring due to virus infection (GLM; *W* = 8.009, *df* = 3, *P* = 0.046) and *Wolbachia* x virus interaction (GLM; *W* = 10.354, *df* = 3, *P* = 0.016). The average fold change in expression between Mel and WT was highest when ZIKV was present.

Expression levels of the putative NF-κB repressing factor (NFKBR, AAEL008415-RA) were significantly affected by *Wolbachia* infection (GLM; *W* = 23.217, *df* = 3, *P* < 0.0001) (Fig 6B), with higher expression levels observed in WT mosquitoes than Mel mosquitoes during all three virus infection treatments (Mann-Whitney U test; DENV-1 –*U* = 57, *P* = 0.0202; ZIKV–*U* = 36.50, *P* = 0.002; ZIKV/DENV-1 –*U* = 43, *P* = 0.0032). In the generalized linear model, there was no significant effect of virus or *Wolbachia* x virus.

Expression levels of the cholesterol and lipid binding protein Niemann-Pick Protein C1b (NPC1b, AAEL009531-RA) were significantly affected by *Wolbachia* (GLM; *W* = 5.374, *df* = 3, *P* = 0.002), virus (GLM; *W* = 12.50, *df* = 3, *P* = 0.006) and *Wolbachia* x virus (GLM; *W* = 26.226, *df* = 3, *P* < 0.0001) (Fig 6C). Expression was significantly higher in Mock-infected Mel mosquitoes than WT mosquitoes (Mann-Whitney U test; *U* = 14, *P* = 0.0096). However, expression levels were significantly lower for Mel mosquitoes in all treatments where virus was present, compared to the Mock-infected Mel group (DENV-1 –*U* = 12, *P* = 0.0015; ZIKV–*U* = 27, *P* = 0.0171, ZIKV/DENV-1 –*U* = 36, *P* = 0.0467).

Expression levels of the putative pupal cuticle protein (PCP, AAEL011045-RA) were significantly affected by *Wolbachia* (GLM; *W* = 12.659, *df* = 3, *P* < 0.0001), but not by virus, or *Wolbachia* x virus interaction in the GLM (Fig 6D). Pairwise comparison revealed that Mel mosquitoes had significantly higher levels of PCP expression than WT mosquitoes during infection with DENV-1 (Mann-Whitney U test; DENV-1 –*U* = 30, *P* = 0.0006; ZIKV/DENV-1 –*U* = 57, *P* = 0.0357).

Expression levels of the iron-binding protein transferrin 1 (TSF1, AAEL015458-RA) were significantly affected by *Wolbachia* (GLM; *W* = 91.826, *df* = 3, *P* < 0.0001), virus (GLM; *W* = 8.747, *df* = 3, *P* = 0.033), and *Wolbachia* x virus (GLM; *W* = 10.288, *df* = 3, *P* = 0.016) according to the GLM (Fig 6E). In Mock-infected mosquitoes, TSF1 levels were higher for Mel than WT (Mann-Whitney U test; *U* = 8.5, *P* = 0.0008). When any virus was introduced, WT TSF1 expression dropped greatly (Mann-Whitney U test; WT Mock vs; DENV–*U* = 14, *P* = 0.0003; ZIKV–*U* = 7, *P* = 0.0001; ZIKV/DENV-1 –*U* = 20, *P* = 0.0023) while Mel TSF1 expression levels with any virus were not significantly different to TSF1 levels with Mock infection (Mann-Whitney U test; Mel Mock vs; ZIKV–*U* = 67.5, *P* = 0.8972; DENV–*U* = 46, *P* = 0.1672; ZIKV/DENV-1 –*U* = 58, *P* = 0.3596).

## Discussion

### Effect of co-infection on pathogen blocking

Biological control programs involving *Wolbachia*-infected mosquitoes must be sufficiently robust to restrict virus transmission in epidemiologically complex environments. There is concurrent transmission of multiple arboviruses in many areas of the world, with *A*. *aegypti*, a major vector of DENV, CHIKV, YFV and ZIKV, capable of transmitting multiple viruses in a single bite [59, 60]. Prior to our study, there was no information on whether *Wolbachia* was capable of inhibiting infection with two pathogens in a mosquito at the same time. Given that different arboviruses induce different immune responses in mosquito hosts, and exhibit different infection dynamics in their hosts [65, 73], we sought to determine if *Wolbachia* would be less effective at blocking two viruses when they were fed simultaneously. We examined disseminated infection in ZIKV/DENV-1 and ZIKV/DENV-3 co-infected *A*. *aegypti* heads/thoraces with and without infection with the *w*Mel *Wolbachia* strain, using the same mosquito line being released into the field in Brazil.

Our data indicate that the ZIKV, DENV-1, and DENV-3 strains that we used, which were all isolated in Brazil in the last five years, were capable of infecting wildtype *A*. *aegypti* mosquitoes. These viruses exhibited different infection dynamics, varying in terms of prevalence of infection, and viral load. We observed that, as expected, *w*Mel-infected mosquitoes challenged with any of these viruses were significantly less susceptible to infection than WT mosquitoes. While those Mel mosquitoes that did become infected exhibited lower viral titres, even at 21dpi. These data were reflective of previous studies on pathogen blocking in *w*Mel-infected *A*. *aegypti*, which indicate that for this strain strong pathogen blocking occurs against a range of viruses [36–38], although it is slightly less effective at blocking arbovirus infections than other *Wolbachia* strains [26, 28, 40].

During the co-infection assays, mosquitoes were fed a blood meal containing fresh ZIKV/DENV-1 or ZIKV-DENV-3 in approximately equal proportions, with quantification via RT-qPCR taking place immediately after the virus was harvested from infected cell culture. In these experiments we observed that Mel mosquitoes challenged with two viruses still exhibited significantly lower prevalence of infection than WT mosquitoes–in terms of overall number of mosquitoes infected by at least one virus. For those few Mel mosquitoes that did become infected with any virus, we observed that the viral load was still significantly lower than for WT mosquitoes.

We observed that the prevalence of infection and viral load in co-infected Mel mosquitoes was not significantly higher than for mono-infected Mel mosquitoes, suggesting that the ability of *w*Mel to block virus infection was not significantly impacted by the presence of an additional virus. We did observe a significant difference between mono-infected and co-infected groups for two conditions, however in both of these cases the effect was decreased prevalence of infection associated with the co-infection. These statistical comparisons were performed with awareness of the fact that only one mono-infection experiment was conducted for each virus, compared to two experiments each for ZIKV/DENV-1 co-infection, and for ZIKV/DENV-3 co-infection. However, given that the data for the mono-infection assays revealed a similar level of pathogen blocking to previously published data from our group with the same *Wolbachia* strain [38, 49], we would not expect the results of the comparisons to change greatly if additional experiments had been performed.

We also examined the effect of *Wolbachia* infection on virus transmission in ZIKV/DENV-3-challenged mosquitoes, by collecting their saliva and then injecting it into naïve mosquitoes, in an experiment akin to a plaque assay in live mosquitoes [38, 42]. Saliva samples were collected at 14 and 21dpi, and then injected into a group of mosquitoes, a proportion of which were assayed via RT-qPCR for the presence of virus. Mel saliva sample selection was intentionally biased, as the saliva from the five head/thorax samples (out of 40 across the two time-points) that were positive for at least one virus were selected for injection. This allowed us to determine whether transmission of these viruses was possible if Mel mosquitoes had detectable disseminated infection. As such, our data likely underestimate the degree to which *w*Mel would inhibit viral transmission in a mosquito population. In spite of this bias, at both 14 and 21dpi, we observed a significantly lower overall prevalence of infection in the mosquitoes injected with saliva from Mel mosquitoes, with only 5/16 Mel saliva positive for any virus, as opposed to WT saliva where 15/16 were positive for any virus. Only 1 Mel saliva sample produced ZIKV/DENV-3 co-infection after injection, as opposed to 9/16 for WT saliva.

These results suggest that while the prevalence of disseminated infection is greatly reduced, a small number of Mel mosquitoes that do get infected may still be able to transmit virus, although the overall transmission rate is likely to be much lower than for WT mosquitoes. Virus transmission in a small subset of *w*Mel-infected mosquitoes has previously been described [43, 44], and is not expected to greatly impact the ability of a *w*Mel-infected *A*. *aegypti* population to limit virus transmission, as the fact that most individuals in the population appear to be refractory to infection would be sufficient to collapse transmission [44]. Taken together, our data indicate that pathogen blocking is still highly effective in *w*Mel-infected *A*. *aegypti* that are challenged with two arboviruses at the same time, and we have found no evidence to suggest that this effect would not occur for mosquitoes in the field.

We observed lower overall prevalence in mosquitoes after infection with the DENV-3 isolate, compared to the DENV-1 and ZIKV isolates that we used. Similarly, DENV-3 load in mosquitoes was lower than what was seen with the other two viruses, and plaque assays revealed that it also had less infectious virus. These data were in line with previous experimental infection assays we have published using this isolate [49]. The results suggest that the DENV-3 MG20 strain is likely to be less infectious to mosquitoes than the other viruses. Several previous studies have indicated that there can be great variability in infectivity between DENV isolates in mosquitoes, and predominant underlying factors in this variation include DENV serotype, and viral genetic lineage [44, 74]. An important factor to note is that we still observed strong *Wolbachia*-induced interference during ZIKV/DENV-1 where the titre of both viruses was high.

### Changes in viral infection dynamics during co-infection

Our data revealed several potential examples of viral competition during co-infection in wildtype mosquitoes. We saw that co-infection led to decreased prevalence of ZIKV and DENV-1, but not DENV-3. Additionally, viral load during co-infection was lower than viral load during the mono-infection assays at least one time point for all three viruses. We also observed higher prevalence of DENV-1 than ZIKV during ZIKV/DENV-1 co-infection, and higher prevalence of ZIKV than DENV-3 during ZIKV/DENV-3 co-infection. Other studies on arbovirus co-infection have found similar results. This includes studies on mosquitoes that found higher ZIKV load during ZIKV/DENV-2 co-infection [59], reduced DENV-2 dissemination at 7dpi during DENV-2/CHIKV co-infection [75], or a competitive advantage for DENV-4 during DENV-1/DENV-4 co-infection [76], and also reduced growth of ZIKV during ZIKV/CHIKV infection of mammalian cells [77]. Taken together with our data, these studies suggest that there is no one virus always has a competitive advantage over another. Instead it is more likely that the dominance of one virus during co-infection occurs due to factors associated with specific viral isolates, with the mosquito lines used, or potentially due to factors associated with the virus culturing or infection conditions that are used. Further analysis of the nature and mechanisms that underlie competitive relationships between different viruses in mosquitoes is worthy of future study.

We observed several instances where the overall prevalence of viral infection in Mel mosquitoes was lower with co-infection than with mono-infection. As this pattern was not consistent across all conditions, it is our opinion that these differences likely reflect stochastic variation between experimental infections, rather than reflecting decreased infectivity due to co-infection. So few *Wolbachia*-infected mosquitoes became infected with any virus that it was difficult to make any definitive statement on changes in viral dynamics due to co-infection. In the one instance where we observed a significant difference in a comparison (where DENV-1 levels were lower during ZIKV/DENV-1 co-infection than for mono-infection), there were only 3 positive Mel samples. Across all experiments, we only observed 4 co-infected Mel mosquitoes, all for ZIKV/DENV-1. Each of these samples had higher levels of ZIKV than DENV, exhibiting the same overall trend towards higher ZIKV load seen with the WT mosquitoes. While the number of virus-infected Mel mosquitoes was too low to make a definitive statement on whether co-infection and *Wolbachia* affect virus dynamics, our findings reveal a clear lack of evidence that co-infection with these viruses could produce less effective pathogen blocking.

### *Wolbachia* x virus effects on gene expression

In order to gain insight into the impacts of *Wolbachia* and virus infections on the mosquito host at the transcriptional level, we quantified the expression of five genes associated with viral infection at 24 hours post-infection with DENV-1, ZIKV or ZIKV/DENV-1. Pathogen blocking has been associated with the upregulation of antimicrobial peptides, including, prominently, *defensin c* (DEFC) [47–49]. Knockdown of DEFC via RNAi led to less effective pathogen blocking against DENV [47, 50]. We observed consistently higher levels of DEFC in Mel mosquitoes than in WT in all treatments, and a higher average fold change in expression for Mel mosquitoes infected by ZIKV in mono- or co-infection. The specific antiviral activity of DEFC remains unknown, but our results provide additional evidence of a role in pathogen blocking.

We assessed levels of the cholesterol binding/transport protein NPC1b, and determined that while Mel mosquitoes had significantly higher expression levels than WT in the Mock treatment, there was no consistent effect of *Wolbachia* in the virus-infected treatments. NPC1b has previously been shown to promote DENV infection in *A*. *aegypti* [64], while cholesterol has been linked to pathogen blocking in *Wolbachia*-infected *Drosophila* and mosquitoes [54, 55]. It is possible that assessing NPC1b levels at a later time post-feeding may provide further insight into a potential role for this gene in pathogen blocking.

We assayed expression levels of a pupal cuticle protein (PCP) that is downregulated during DENV infection, and binds WNV (*West Nile Virus*) envelope protein, thereby inhibiting infection [65]. Interestingly, we observed significantly higher PCP levels in Mel mosquitoes than WT mosquitoes when DENV-1 was present during either mono- or co-infection, suggesting that PCP may plays some role in *Wolbachia*-induced response to DENV-1. However, we saw no effect of *Wolbachia* on PCP expression during ZIKV mono-infection.

AAEL008415-RA, a putative NF-κB repressing factor (NFKBR) was identified from microarray studies of *A*. *aegypti* as being significantly downregulated by both *w*Mel and *w*MelPop infection [48], and strongly upregulated during WNV and DENV infection [65]. Comparative analysis of protein sequences in Uniprot BLAST (http://www.uniprot.org/blast/) suggested that this protein had very high levels of similarity to NFKB repressors across many species. We observed significantly higher transcription levels of NFKBR in all WT virus-infected treatments compared to Mock-infected WT mosquitoes, and in comparison to Mel mosquitoes challenged with any virus. A potential explanation for this is that in WT mosquitoes, virus infection induces expression of NFKBR and thus helps to supress NFKB transcriptional activity, which is known to impact DENV infection [78, 79]. For Mel mosquitoes there was no virus-induced increase in NFKBR expression, potentially suggesting that *Wolbachia* infection limits viral induction of this gene.

We observed *Wolbachia* and virus-specific induction of the iron-binding gene TSF1. Levels of this gene have been shown to be strongly induced during *Wolbachia* infection [46, 48], with this effect responsive to changes in host nutritional status [49]. Iron sequestration is thought to represent a key component of the mosquito immune response to many different pathogens [80–83], while iron is also essential for *Wolbachia* in many different host species [84–86]. We observed that Mel mosquitoes had higher TSF1 levels under mock infection conditions. During infection with DENV and/or ZIKV, TSF1 levels in WT mosquitoes were greatly decreased, while TSF1 levels in Mel mosquitoes were increased beyond levels in the Mock treatment. This suggests that TSF1 is highly and differentially responsive to *Wolbachia* and virus infection, and indicates that iron binding may be an important aspect of the pathogen blocking response.

### Limitations of the study

During the oral infection assays, we assessed viral titre via two different methods, and obtained drastically different results. The initial titration in the infectious blood meal provided to mosquitoes was based on RT-qPCR quantification for viral genomic RNA, while the second was via plaque assay. It should be noted that these assays measure viral titre differently, with the plaque assay describing the titre of infectious virus after 6 days of infection in mammalian cells and the RT-qPCR performed directly on the virus-infected cell culture supernatant that was used in the oral infection experiments. Our plaque assay data (Table 1) depict a high degree of difference in viral load between the ZIKV, DENV-1, and DENV-3 isolates. It should be noted that these estimates of viral load were determined for undiluted virus-infected supernatant, and are therefore not reflective of the virus mixture that fed to our mosquitoes. Critically, both techniques have inherent difference in accuracy and sensitivity, and therefore provide different estimates of viral titre, and there is no guarantee that either method provided an accurate estimate of the number of particles of either virus that reached the mosquito midgut epithelium. We were also limited in that we were only able to perform a single experiment with ZIKV, DENV-1 and DENV-3 mono-infections. While our results showed a similar of *Wolbachia*-induced blocking to what we had seen in previous studies with the same viruses [33, 38, 42], it limited our ability to compare data between the mono- and co-infection datasets.

Another limitation of our study occurred due to the strong nature of the pathogen blocking seen in the Mel mosquitoes. Overall, too few Mel mosquitoes were positive for both viruses after co-infection, making it impossible for us to determine whether there were *Wolbachia* x co-infection interactions that affected virus dynamics similar to what we observed with wildtype mosquitoes. A further limitation of the study was that additional DENV and ZIKV isolates were not examined. We tested isolates from only two of the four DENV serotypes, although this did include an isolate of DENV-1, which has previously been shown to induce weaker blocking in *w*Mel-infected mosquitoes [44]. We also examined only a single ZIKV isolate, although of the two isolates that were available to us, we did select the isolate that displayed stronger infection in mosquitoes [38]. Finally, the gene expression assays were limited by the fact that we did not have the resources to test additional genes, transcriptional changes in the presence of our DENV-3 isolate, to perform a broader transcriptomic assay at multiple time points, or to perform gene silencing assays in order to assess the role of each gene in pathogen interference. As with all assays of gene expression, it is important to remember that changes in transcript levels do not always correlate to changes at the protein level. Consequently, we recommend further validation of our five gene candidates.

## Conclusions

Here we present the first examination of arbovirus co-infection in *Wolbachia*-infected mosquitoes. We have demonstrated that the *w*Mel *Wolbachia* strain is capable of blocking ZIKV, DENV-1 and DENV-3 when challenged singly or in co-infection, with Mel mosquitoes demonstrating significantly reduced prevalence of infection and viral load compared to wildtype mosquitoes. Only a small minority of *w*Mel-infected mosquitoes assayed across multiple time points and experiments, demonstrated infection with both viruses simultaneously. In contrast, wildtype mosquitoes displayed high levels of disseminated co-infection. We also demonstrated that wildtype mosquitoes were capable of transmitting both ZIKV and DENV-3 at high rates after co-infection, however transmission rates for Mel mosquitoes were greatly reduced, even after we intentionally biased our data by selecting saliva from the small proportion of Mel mosquitoes that had detectable disseminated infection. Our data provide a strong indication that co-infection would not adversely affect pathogen blocking in the *w*Mel-infected *A*. *aegypti* that are currently in the field in Rio de Janeiro, Brazil. Given that this region also sees regular transmission of CHIKV and YFV, in addition to DENV and ZIKV, examination of the ability of *Wolbachia* to block infection with these viruses would be beneficial. It would also be interesting to examine the effect of super-infection of two arboviruses, feeding one after the other has already established infection in the mosquito, and to assess whether *Wolbachia*-infected mosquitoes in the field respond to co-infection in a similar manner. However, our data provide no evidence that either of these scenarios would detrimentally impact on pathogen blocking, or the utility of *Wolbachia* as an arbovirus control agent.

## Supporting information

S1 File List of primers & probes. (DOCX)

S2 File Infection data. (XLSX)

S3 File Saliva injection data. (XLSX)

S4 File Gene expression data. (XLSX)
