# Supplementary material for: Pathogen blocking in Wolbachia-infected Aedes aegypti is not affected by Zika and dengue virus co-infection
Source: PLoS Negl Trop Dis. 2019 May 20;13(5):e0007443. doi: 10.1371/journal.pntd.0007443 (PMC6544317; doi:10.1371/journal.pntd.0007443)
Supplement: S1 File — (DOCX) [file pntd.0007443.s001.docx]

**S1 File - List of Primers and Probes**

| **Target** | **Gene ID** | **Primers** | **Reference** |
| --- | --- | --- | --- |
| RpS17 | AAEL004175-RA | Virus Quantification  F - 5’-TCCGTGGTATCTCCATCAAGCT-3’  R - 5’-CACTTCCGGCACGTAGTTGTC-3’  Probe - 5’-/5HEX/CAGGAGGAG/ZEN/GAACGTGAGCGCAG/3lABkFQ/-3’  Gene expression  F - 5’-CACTCCCAGGTCCGTGGTAT-3’  R - 5’-GGACACTTCCGGCACGTAGT-3’ | [1,2] |
| Zika virus |  | F - 5’-TTGGTCATGATACTGCTGATTGC-3’  R - 5’- CCTTCCACAAAGTCCCTATTGC-3’  Probe - 5’-FAM/CGGCATACAGCATCAGGTGCATAGGAG/BHQ1-3’ | [3] |
| Dengue virus |  | F - 5’-AAGGACTAGAGGTTAGAGGAGACCC-3’  R - 5’-CGTTCTGTGCCTGGAATGATG-3’  Probe - 5’-/TEX615/AACAGCATATTGACGCTGGGAGAGACCAGA/3IAbRQSp/3’ | [4] |
| Defensin C | AAEL003832-RA | F -5'-TTGTTTGCTTCGTTGCTCTTT-3'  R - 5'-ATCTCCTACACCGAACCCACT-3' | [5] |
| NF-kappaB repressing factor, putative  (Currently unannotated) | AAEL008415-RB | F - 5'-GGGATAGTTGCAACGTGGAG-3'  R - 5'-CCTCTTTGAGTTTGTCCTTTG-3' | Designed in this study |
| Niemann-Pick Protein C1b | AAEL009531-RA | F - 5'-ACACCTTTTGCGAATCCTGCCC-3'  R - 5'-CATGGACGTTCAGATGACCGGC-3' | [6] |
| Pupal cuticle protein, putative | AAEL022261-RA, (formerly AAEL011045-RA) | F - 5'-GGTGTCGTCCCATTGGCC-3'  R - 5'-CGGCTCTCGACCTGGGATTTC-3' | [7] |
| Transferrin 1 | AAEL015458-RA | F - 5'-GTTCCGGTACAACCTGGAGA-3'  R - 5'-TTCAGCTCGATCAGGGAAGT-3' | [8] |

**References:**
